# The association between alcohol, marijuana, illegal drug use and current use of E-cigarette among youth and young adults in Canada: results from Canadian Tobacco, Alcohol and Drugs Survey 2017

**DOI:** 10.1186/s12889-019-7546-y

**Published:** 2019-09-02

**Authors:** Vrati M. Mehra, Asvini Keethakumar, Yvonne M. Bohr, Peri Abdullah, Hala Tamim

**Affiliations:** 1 School of Kinesiology and Health Sciences, Faculty of Health, York University, Toronto, Canada; 2 Department of Psychology, York University, 4700 Keele Street, Toronto, Canada

**Keywords:** E-cigarette, Vaping, Illicit drugs, Drugs, Youth, Young adults, Smoking, Alcohol, Canada

## Abstract

**Background:**

E-cigarettes have grown in popularity around the world since 2003. Although marketed as a smoking cessation tool, e-cigarettes can lead to tobacco cigarette smoking in youth. In Canada, among all age groups, youth and young adults have the highest prevalence of e-cigarette use. The objective of this study was to assess the factors associated with e-cigarette use among youth and young adults in Canada, and to specifically examine the association between alcohol, marijuana and illicit drug use.

**Methods:**

Data from the 2017 Canadian Tobacco, Alcohol and Drugs Survey were used. The sample was restricted to those aged 15–24 years (*n* = 10,322), and main outcome defined as ‘E-cigarette use in the past 30-days’. Multivariable logistic regression was performed to assess the association between the main outcome and substance use variables (illicit drug, marijuana and alcohol use), tobacco exposure variables, and demographic and health-related factors.

**Results:**

6.2% Canadians aged 15–24 reported using e-cigarettes in the past 30-days, while 23.9% reported having ever tried e-cigarettes. Twenty-three percent of the past 30-day users reported using e-cigarettes every day and 72.5% of the past 30-day users reported having nicotine in their last e-cigarette. Additionally, youth aged 15–17 were 4.95 times more likely to be e-cigarette users as compared to those aged 22–24 (OR: 4.95, 95% CI: 3.1–7.9). Moreover, e-cigarette use was significantly associated with marijuana use (OR:4.17, 95% CI: 2.6–6.7) and alcohol use (OR: 5.08, 95% CI: 2.9–9.0), and approached significance with illicit drug use (OR: 1.68, 95% CI: 1.0–2.9). Furthermore, being a current smoker (OR: 2.93, 95% CI: 1.8–4.7) and male (OR: 2.28, 95% CI: 1.5–3.4) was significantly associated with the outcome.

**Conclusion:**

This study is nationally representative and provides insight into e-cigarette use among youth and young adults aged 15–24 years. Given that e-cigarettes can be used as illicit drug delivery systems, more studies are needed to understand how Canadian youth and young adults are using e-cigarettes. Stricter restrictions on public e-cigarette smoking, and awareness campaigns informing youth of risks of e-cigarette smoking should be implemented.

## Background

Since their invention in 2003, electronic cigarettes, also known as e-cigarettes, vapes and vape pens, have consistently grown in popularity across the globe [1]. In 2016, the e-cigarette market earned more than 8 billion dollars in profits globally. This amount is expected to go up to 26 billion dollars by the year 2023 [2]. E-cigarettes were originally marketed as a cleaner and safer alternative to the traditional combustible cigarette [3]. However, since incorporating a wide variety of flavours, designs, nicotine levels, and drug delivery options, the e-cigarette market has grown rapidly to appeal to a large demographic of consumers [3–5].

Although media portray the e-cigarette as an ideal alternative to smoking, the benefits and harms of e-cigarette use remain highly debated in the research community [6–8]. Some studies report the use of e-cigarettes as an effective smoking cessation tool, whereas others argue that it can lead to the introduction of tobacco cigarette smoking [6, 7, 9]. Additionally, e-cigarettes have been associated with a decline in overall health, including lung tissue damage, worse cardiovascular health outcomes and increased inflammatory responses [10–12]. Although none in Canada, some international studies have also shown positive associations between risky behaviors such as the use of illicit drugs, marijuana, alcohol and e-cigarette use [13–22]. Consequently, increased concerns have arisen among substance use researchers since recent e-cigarette designs have grown to accommodate illicit substances. A study conducted by Breitbarth (2018) utilizing online illicit drug forums reported that many illicit drugs, including methamphetamine, heroin, cocaine, fentanyl, have the potential to be vaporized via an e-cigarette, a practice gaining traction among drug users [5].

Up until 2017, the sale of e-cigarettes containing nicotine was considered illegal in Canada, but non-nicotine e-cigarettes were legal and widely available to the public [23]. However, in an effort to reduce the consumption of traditional cigarettes, nicotine vaping alternatives were legalized by the Canadian government in 2018 under the Tobacco and Vaping Products Act (TVPA) for adults over the age of 18 [24]. The TVPA also placed restrictions on the use of marketing practices such as interesting shapes and flavors, that make e-cigarettes attractive to younger populations, in order to limit their appeal to youth [25]. Given the strong association between factors such as fruit and candy flavourings [26, 27] and ‘sleek’ designs of e-cigarettes [28] on youth and young adults’ motivation towards smoking e-cigarettes, such restrictions should prove beneficial in the coming years.

In Canada, the ever and past 30-day use of e-cigarettes has consistently increased over the past few years. From 2013 to 2015, the ever use of e-cigarette increased from 9 to 13% in the overall population aged 15 years and older, while the past 30-day use increased from 2 to 3% [29, 30]. More importantly, during the same time period, the rates of past 30-day use of e-cigarettes almost doubled for youth and young adults (3% in youth aged 15–19 in 2013 to 6% in 2015; 4% in young adults aged 20–24 to 6% in 2015), and rates of ever using e-cigarettes increased by 30% in youth aged 15 to 19 and 50% for young adults aged 20 to 24. [29, 30].

Despite the popularity of e-cigarettes among youth and young adults, there is a lack of nationally representative studies on its use within those age groups. Additionally, there is limited awareness surrounding the concomitant use of illicit drugs and e-cigarettes in young Canadians. Given the concrete relationship between the use of illegal substances and the traditional tobacco cigarettes [31, 32], it is important to know whether similar associations exist between illegal substance use and e-cigarettes. Therefore, the objective of the present study is to confirm the prevalence and assess the characteristics associated with e-cigarette use among youth and young adults in Canada, with a specific focus on its association with substances such as illicit drugs, marijuana and alcohol.

## Methods

### Study design and participants

The secondary data analysis for this study was based on the 2017 version of the Canadian Tobacco Alcohol and Drugs Survey (CTADS), sponsored by Health Canada and conducted by Statistics Canada every 2 years. The CTADS is a national cross-sectional survey that aims to collect data on tobacco, alcohol, and drug use, and contribute knowledge on risky behaviours within the Canadian population, with a specific focus on younger populations. For the 2017 cycle of CTADS, data collection was initiated on February 1st, 2017 and ended on December 31st, 2017. The target population consisted of individuals aged 15 and above and residing in the 10 Canadian provinces. Full-time residents of institutions and those living in the three territories (Yukon, Northwest Territories and Nunavut) were not included in the survey. The survey was conducted using a specialized sample design which included a two-phase stratified random sampling of telephone numbers. Those without telephones or cellphones were not recruited for the study, however survey estimates have been applied to include those without landlines and cellular phones. Participation in the survey was voluntary and data were collected directly from the survey respondent. Additionally, missing or inconsistent information was handled by Statistics Canada through various types of editing to ensure logical relationship between responses. A more detailed breakdown of the survey’s design and sampling procedure can be found on the Statistics Canada website [33]. For the purposes of this study, only individuals who reported being 15–24 years of age were included. The total sample comprised of 10,322 individuals, representing approximately 4,443,600 Canadians of ages 15–24. The average age of the respondents was 19.5 years, whereas the median age was 20 years. Additionally, 48.6% of the sample were females and 51.4% were males.

### Main outcome

The main outcome of the study was ‘e-cigarette use’. This was measured based on the following question: “In the past 30 days did you use an electronic cigarette, also known as an e-cigarette?” The respondents were provided with the following options, “Yes”, “No”.

### Covariates

The main independent variables for this study included, substance use variables comprising of alcohol use in the past 12 months (asked by the question, ‘How often did you drink alcoholic beverages during the past 12 months’ with ‘Never’ recoded as ‘no’ and any alcohol use recoded as ‘yes’), marijuana (asked by the question ‘During the past 12 months have you used marijuana?’ yes or no), and illicit drug use (including cocaine, speed/meth, ecstasy, hallucinogens, salvia, heroin, inhalants, abuse of pain relievers, stimulants and sedatives to get high in the past 12 months, assessed using questions asking participants whether they had used a specific substance in the past 12 months, yes or no). Tobacco use variables included household smoking (asked by the question, ‘Do you/Does anyone in your household smoke cigarettes? Yes or No) current smoking status (variable created by Statistics Canada based on multiple questions), and other tobacco products (comprised of use of either cigarillos, cigars, tobacco water pipe or smoke-less tobacco in the past 30 days, assessed using questions asking participants whether they had used any of the above specified tobacco products in the past 30 days, yes or no). In addition, a wide range of covariates including demographic and health factors, and tobacco exposure variables, were considered. Demographic and health factors included age, sex, province, indigenous status (asked by the question ‘Are you an Aboriginal person, that is, First Nations, Métis or Inuk (Inuit)?’) , community dwelling (rural or urban), current employment (asked by the question ‘Last week, did you work at a job or business?’), and self-perceived health (asked by the question ‘In general, would you say your health is...?’).

Additional variables considered in the analysis also included whether nicotine was present in the last e-cigarette used, frequency of using e-cigarette (everyday, occasionally or not at all) and reasons for using e-cigarettes.

### Statistical analysis

Descriptive statistics of the main outcome and other variables were conducted. Odds Ratios (ORs) and 95% Confidence Intervals (CIs) were obtained using bivariate and multivariable logistic regression. When all the variables were considered, a maximum of 3.16% of the cases had missing information. These cases were excluded from the analysis. Population weights were applied to each calculated estimate and bootstrapping was performed to adjust for the complex sampling methodology [33]. All analyses were conducted using Stata Statistical Software, version 13, (StataCorp, College Station, TX). Statistical significance for all analyses was set at alpha < 0.05.

## Results

The total sample was comprised of 10,322 individuals representing approximately 4,443,600 Canadians aged 15–24 years. Among them, 6.2% reported having used e-cigarettes in the past 30-days, while 23.9% reported having ever tried e-cigarettes. Approximately 3.16% of the respondents did not provide information on at least one of the covariates and were excluded from the regression analysis.

Among the past 30-day users, 23.1% reported using e-cigarettes every day, whereas 57.7% reported using e-cigarettes occasionally (Table 1). Furthermore, 72.5% of the past month users reported having nicotine in their last e-cigarette. Additionally, some of the main reasons for using e-cigarettes in the past 30-days included, “They come in flavors I like” (63.5% past 30-day users and 42.4% ever users), “Curious- want to know how it tastes” (57.7% past 30-day users and 75.5% in ever users), “E-cigarettes are more acceptable to non-tobacco users” (44.9% in past 30-day users and 29.2% in ever users), “They might be less harmful to people around me than cigarettes” (54.8% in past 30-day users and 33.3% in ever users), and “They might be less harmful to me than smoking cigarettes” (54.0% in past 30-day users and 32.8% in ever users). Finally, among past 30-days users, over 49.8% reported getting e-cigarettes from friends and relatives and 32.9% reported getting them from a vape shop or vapor lounge. Other places where e-cigarettes were accessed included convenience store or gas stations, and the internet (6.5 and 6.0% in past 30-day users, respectively).

**Table 1:** Presence of nicotine, frequency of e-cigarette use and reason of using e-cigarettes among ever and past 30-day e-cigarette users aged 15–24 years based on the Canadian Tobacco, Alcohol and Drugs Survey, 2017

	E-cigarette use ever % (N)	E-cigarette use in the past 30 days % (N)
Last e-cigarette contained nicotine		
Yes	51.2% (523,300)	72.5% (196,900)
No	37.8% (385,900)	24.5% (66,400)
Uncertain	11.1% (112,900)	3.1% (8300)
Frequency of using e-cigarette		
Everyday	3.3% (34,100)	23.1% (62,900)
Occasionally	17.1% (175,000)	57.7% (157,400)
Not at all	79.6% (813,100)	19.2% (52,300)
Reason for Using E-cigarette		
Reason for use- They come in flavours I like		
Yes	42.4% (429,900)	63.5% (170,800)
No	57.6% (584,600)	36.5% (98,000)
Reason for use-E-cigarettes are more acceptable to non-tobacco users		
Yes	29.2% (296,000)	44.9% (120,600)
No	70.8% (248,400)	55.1% (148,200)
Reason for use-They might be less harmful to people around me than cigarettes		
Yes	33.3% (337,400)	54.8% (147,200)
No	66.8% (677,200)	45.3% (121,600)
Reason for use- They might be less harmful to me than smoking cigarettes		
Yes	32.8% (331,500)	54.0% (145,200)
No	67.3% (683,100)	46.0% (123,600)
Reason for use- Using e-cigarettes helps people quit smoking cigarettes		
Yes	23.3% (236,000)	50.9% (136,700)
No	76.7% (778,500)	49.1% (132,100)
Reason for use- Curious - want to know how it tastes		
Yes	75.5% (766,000)	57.7% (155,000)
No	24.5% (248,500)	42.4% (113,900)
Reason for use- E-cigarettes don’t smell		
Yes	17.7% (179,200)	29.9% (80,300)
No	83.3% (835,300)	70.1% (188,500)
Where do you usually get the e-cigarettes you use?		
A friend or relative (borrowed, shared or bought)	71.3 (717,200)	49.8 (133,300)
A vape shop or vapor lounge	9.6 (96,600)	32.9 (88,000)
A convenience store or gas station	12.6 (126,900)	6.5 (17,500)
Over the internet	1.9 (18,800)	6.0 (16,100)
Other^a^	4.6 (46,300)	4.8 (12,800)

*N* is the weighted sample size based on the person weights provided by Statistics Canada [33]. Less than 2% of the respondents did not provide information on one or more the variables mentioned above. These cases were excluded from the analysis

^a^Includes: A small kiosk, A supermarket, grocery store or drug store, A smoke shop, tobacco specialty store, outlet store, A bar, pub, restaurant or casino and Other

**Fig. 1:**
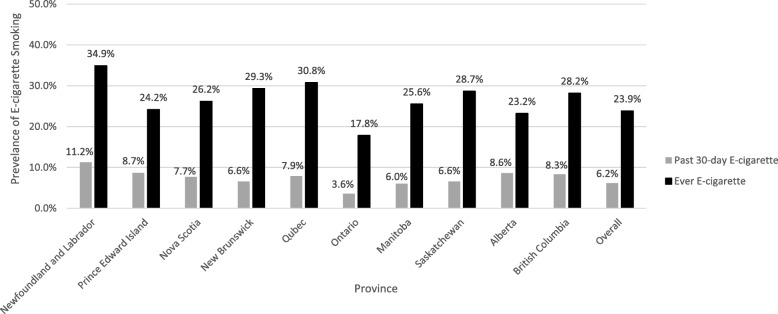
The prevalence of ever e-cigarette use and past 30-day e-cigarette use among 10 provinces of Canada, based on Canadian Tobacco, Alcohol and Drugs Survey, 2017

The prevalence of ever and past 30-day e-cigarette use, for all 10 provinces, are displayed in Fig. 1. Among all provinces, Newfoundland and Labrador had the highest prevalence of ever use of e-cigarettes (34.9%), followed by Quebec (30.8%), New Brunswick (29.3%) and Saskatchewan (28.7%). Newfoundland and Labrador also had the highest prevalence of past 30-day use of e-cigarette (11.2%), followed by Prince Edward Island (8.7%), Alberta (8.6%), and British Columbia (8.3%). Among all provinces, Ontario had the lowest prevalence of both ever and past 30-day use, with prevalence rates of 17.8 and 3.6%, respectively.

Results displayed in Table 2 present the various covariates associated with past 30-day e-cigarette use. Among the *substance use variables*, 25.5% of illicit drug users and 17.3% of marijuana users reported using e-cigarettes in the past 30-days. Additionally, 22.8% of the current smokers within this cohort reported using e-cigarettes in the past 30 days. After adjusting for all other variables (Table 2), illicit drug use approached significance with past 30-day e-cigarette use (OR: 1.68, 95% CI: 1.0–2.4). Additionally, marijuana users were 4.17 times more likely to be e-cigarette users as compared to non-marijuana users (OR: 4.17, 95% CI: 2.6–6.7), as were past year alcohol users, compared to non-alcohol users (OR: 5.08, 95% CI: 2.9–9.0). Among the *tobacco use variables*, those who were from a smoking household were 1.73 times more likely to report e-cigarette use in the past month (OR: 1.73, 95% CI: 1.1–2.6). Additionally, current smokers were more likely to have used e-cigarette in the past 30 days, as compared to those who had never smoked (OR: 2.93, 95% CI: 1.8–4.7), as were other tobacco product users, compared to those non-tobacco product users (OR:1.83 (1.0–3.3).

**Table 2:** Frequencies and unadjusted odds ratios (ORs) of Substance Use, Tobacco Exposure and Use Variables, and Sociodemographic and Health Related Factors, as associated with past 30-day e-cigarette use in individuals aged 15–24 years, based on the Canadian Tobacco, Alcohol and Drugs Survey, 2017

	N (%)	% E-cigarette Use in the past 30-days	E-cigarette Use in the past 30-days Unadjusted OR (95% CI)	*P* Values	E-cigarette Use in the past 30-days Adjusted OR (95% CI)	*P* Values
Substance use (Last 12 months)						
Illicit Drug Use^a^						
No	3,903,700 (91.15)	4.3	1		1	
Yes	378,900 (8.85)	25.5	7.70 (5.2–11.5)	< 0.001	1.68 (1.0–2.9)	0.060
Marijuana						
No	3,166,554 (31.64)	2.0	1		1	
Yes	1,163,700 (68.36)	17.3	10.31 (7.3–14.6)	< 0.001	4.17 (2.6–6.7)	< 0.001
Alcohol						
No	1,248,800 (28.8)	0.9	1		1	
Yes	3,087,300 (71.2)	8.3	10.18 (6.1–17.1)	< 0.001	5.08 (2.9–9.0)	< 0.001
Tobacco exposure variables						
Household smoking						
No	3,419,300 (77.0)	4.0	1		1	
Yes	1,023,200 (23.0)	13.3	3.63 (2.6–5.0)	< 0.001	1.73 (1.1–2.6)	0.010
Current smoking status						
Never	3,748,200 (84.4)	3.2	1		1	
Former	148,900 (3.4)	10.7	4.39 (2.1–9.3)	< 0.001	2.45 (0.9–6.3)	0.065
Current	546,400 (12.3)	22.8	9.84 (7.0–13.9)	< 0.001	2.93 (1.8–4.7)	< 0.001
Other tobacco products^b^						
No	4,125,200 (93.1)	4.8	1		1	
Yes	304,800 (6.9)	24.9	6.94 (4.5–10.8)	< 0.001	1.83 (1.0–3.3)	0.048
Demographic and health related factors						
Age						
15–17	1,389,300 (31.3)	5.9	1.12 (0.8–1.7)	0.573	4.95 (3.1–7.9)	< 0.001
18–21	1,901,700 (42.8)	7.0	1.36 (1.0–1.9)	0.080	2.08 (1.4–3.1)	< 0.001
22–24	1,152,600 (25.9)	5.3	1		1	
Sex						
Female	2,159,500 (48.6)	3.3	1		1	
Male	2,284,100 (51.4)	8.8	2.83 (2.1–3.8)	< 0.001	2.28 (1.5–3.4)	< 0.001
Indigenous status						
No	4,122,600 (82)	6.0	1		1	
Yes	186,400 (18)	9.6	1.68 (1.0–2.8)	0.052	0.78 (0.4–1.6)	0.515
Province^c^						
Western-BC	594,400 (13.4)	8.3	1		1	
Western-Parries	849,900 (19.1)	7.7	0.93 (0.5–1.7)	0.799	0.93 (0.4–2.1)	0.863
Central	2,730,400 (61.5)	5.0	0.59 (0.3–1.1)	0.078	0.63 (0.3–1.3)	0.227
Eastern-Atlantic	268,900 (6.1)	8.1	0.98 (0.5–1.8)	0.945	1.05 (0.5–2.3)	0.911
Community dwelling						
Rural	898,800 (20.2)	7.4	1		1	
Urban	3,544,700 (79.8)	5.9	0.78 (0.5–1.2)	0.266	0.90 (0.5–1.5)	0.691
Current Employment						
Not Employed	1,756,300 (40.62)	4.6	1		1	
Employed	2,567,700 (59.38)	7.1	1.57 (1.1–2.2)	0.010	1.32 (0.9–2.0)	0.188
Self-perceived health						
Very Good/Excellent	3,256,600 (73.3)	5.1	1		1	
Good	998,600 (22.5)	8.7	1.76 (1.3–2.5)	0.001	1.26 (0.8–1.9)	0.265
Fair/Poor	188,100 (4.2)	10.1	2.07 (1.1–3.8)	0.017	1.20 (0.5–2.7)	0.668

*N* is the weighted sample size based on the person weights provided by Statistics Canada [33]. Approximately 3.16% of the respondents did not provide information on one or more the variables. These cases were excluded from the regression analysis

^a^Includes: cocaine, speed/meth, ecstasy, hallucinogens, salvia, heroin, inhalants, abuse of pain relievers, stimulants and sedatives to get high in the past 12 months

^b^Cigarillo, Cigar, Tobacco Water-pipe and Smokeless tobacco

^c^Eastern Atlantic: Newfoundland & Labrador, Nova Scotia, Prince Edward Island & New Brunswick; Central: Quebec & Ontario; Western Prairies: Manitoba, Saskatchewan, & Alberta; and Western British Columbia: British Columbia

Among the *demographic and health factors*, those who were aged 15–17 were 4.95 times more likely than there 22–24 year old counterparts to have used e-cigarettes in the past 30 days (OR: 4.95, 95% CI: 3.1–7.9). Similarly, 18–21 were a little over two times more likely (OR: 2.08, 95% CI: 1.4–3.1) to be e-cigarette users as compared to those aged 22–24. Additionally, males were 2.28 times more likely to be e-cigarette users compared to females (OR: 2.19, 95% CI: 1.5–3.4).

## Discussion

This study sheds light on the prevalence of and factors associated with e-cigarette use in youth and young adults in Canada. In 2017, the national prevalence rate for past 30-day e-cigarette use among this population was 6.2%. This finding is similar to reports from the 2015 CTADS survey (6%) [30], however greater than the 2013 results which suggested less than 4% past 30-day e-cigarette use in this demographic [29]. The prevalence of ever and past 30-day use of e-cigarette was highest in the province of Newfoundland and Labrador, with the lowest reported prevalence in Ontario. Among other factors, this study also shows that using marijuana and alcohol, being a current cigarette smoker, and being male are significantly associated with past 30-day e-cigarette use. Moreover, illicit drug use approached significance with past 30-day e-cigarette use.

After adjusting for *socio-demographic and health related factors, tobacco use variables, and substance use variables*, the use of e-cigarette in illicit drug users was significantly higher than non-drug users. Due to the lack of studies on the dual use of illicit drugs and e-cigarettes in Canada, it is not possible to compare these results to previous Canadian data to assess trends. However, some international studies have shown findings similar to those in the present study [15–17, 34]. Additionally, even fewer studies have explored the potential use of e-cigarettes as illicit drug delivery systems. Blundell et al. (2018), using convenience samples, and Breitbarth et al. (2018), using illicit drug forums, found that drugs such as methamphetamine, ecstasy, cocaine, heroin and fentanyl have the potential to be smoked through e-cigarettes and come with the added advantage of losing their characteristics odours, making them easier to disguise in public settings and from police officials [5, 18]. Once explored further, these findings would warrant stricter rules around smoking e-cigarettes in public settings, as dual delivery has the potential of exposing the public to nicotine smoke as well as illicit drugs. Having said that, more research is needed in the area of substance use via alternative drug delivery systems, in order to clearly understand the mechanisms of their use as well as the risks posed by e-cigarettes.

The present study also showed that marijuana users were significantly more likely to be past 30-day e-cigarette users, compared to non-users. Some studies suggest that vaping of cannabis extracts is becoming more common among marijuana users [19, 35, 36] due to its perception of being less harmful than the traditional burning to smoke methods [4], being easier to disguise due to reduced odours [35], and stimulating less throat irritation upon smoking [37]. Additionally, it is important to note that the current study utilizes results from a survey conducted in 2017, when cannabis was still considered an illegal substance in Canada. In October of 2018, the Cannabis Act legalized the possession and use of cannabis across the nation. This may have implications for the association between marijuana and e-cigarette use in the coming years, and the authors predict that this association will become exacerbated with time. Finally, the association between using alcohol and e-cigarettes has also been noted in other studies [20–22]. However, it is unclear what underlies this association. Hershberger et al. (2016) suggests that the combined use might exist to offset the sedating effects of alcohol via nicotine, while increasing the dopaminergic reward system stimulation through the combination of both substances [38]. While substance use and e-cigarette use were common in the current study, more studies are required to assess the temporal order of these two behaviors.

Among the *tobacco exposure and use variables*, those who had one or more smokers in their household were significantly more likely to have used e-cigarette in the past 30-days, as compared to those belonging to non-smoking households. This is in line with the longitudinal study conducted by Gorini et al. (2016), which found that children and adolescents who belonged to smoking households were two times more likely to become cigarette smokers as young adults, as compared to those who belonged to smoke free households while growing up [39]. It is then possible that witnessing smoking behaviors by close family members might normalize smoking related behaviours, leading to an increased uptake of e-cigarette smoking among younger populations [40]. Furthermore, it is also plausible that exposure to second hand smoke may itself play a role in uptake of e-cigarettes [41]. Those who reported being current tobacco smokers were significantly more likely to be e-cigarette users. These results were expected and are similar to other studies that have found that the combined use of the traditional cigarette and e-cigarettes is common among adolescents and young adults [42–44]. Although, the reasons for the combined use of traditional cigarettes and e-cigarettes are unknown in this study, a study conducted by Kong et al. (2015) among young adults and adolescents, found that commonly stated reasons for using e-cigarettes along with traditional cigarettes included their affordability, the belief that they had reduced health risks, availability of different flavours and the ability to use them in different settings [45].

Among the *demographic and health related variables,* youth aged 15–17 and those aged 18–21 were significantly more likely to be past 30-day e-cigarette users as compared to those aged 22–24. Findings from Reid et al. (2015) were consistent with our findings and also showed that prevalence of e-cigarette was the highest among young Canadians, particularly between the ages of 15–19 and reported that this association subsequently decreased with increasing age [46]. Similar trends have also been seen in the United States. A study by Cullen et al. (2018) reported one in every five high school students in United States had tried e-cigarettes in the past 30 days in 2018. Following such reports, the U. S Surgeon General, Jerome Adams, declared e-cigarette use among youth an ‘epidemic’ in the country and pushed for higher officials to take action [47]. Adolescents’ perceptions of e-cigarettes’, especially flavored e-cigarettes, as being less harmful and not containing nicotine, combined with a greater propensity of experimentation and risk taking behaviors associated with adolescence, may explain the higher rates of e-cigarettes use among minors seen in this study [27, 48]. Among other sociodemographic and health related factors, males were more likely to have used e-cigarettes in the past 30-days as compared to females. Many studies have also shown similar results [21, 42, 49]. While the reasons for this finding could be multifaceted, a study conducted in the United States by Coleman et al. (2015) found that males were almost twice more likely than females to report openness to smoking [50], which when combined with greater social acceptability of e-cigarette smoking [51] might give some insight into why males have greater odds of being e-cigarette smokers when compared to females. However, future research efforts should be directed towards understanding the motivations towards e-cigarette smoking among both sexes.

The present study has several strengths. To our knowledge this is the first study to look at illicit drug, marijuana and alcohol use and their association with e-cigarette use among Canadians aged 15–24. The results of this study are nationally representative and can be used by future studies to assess and track changes in e-cigarette use. Given the changing landscape of e-cigarette use in Canada, multifaceted factors might play a role in its uptake by the Canadian youth. The comprehensive list of covariates used in this study will help deconstruct the complex nature of e-cigarette use and its associated factors within the younger Canadians and aid in the development of specific and targeted policies around its use. Although novel, this study had a few limitations. Due to the design of the survey, important demographic variables such as socioeconomic status and ethnicity were not reported in this study. There was also scarce information on the use of e-cigarettes for smoking illicit drugs included under the illicit drugs category, therefore the authors were unable to examine how many illicit drug users were using e-cigarettes to smoke illegal substances. Additionally, the exclusion of the three territories from the survey may have affected the representation of some indigenous people in this study. As of the 2016 Canadian Census, 6.8% of the Inuit population, 0.8% of the Métis population and 2.1% of the First Nations population in Canada resided in these territories [52]. As frequently known for all cross-sectional studies, the design of this study prevents any causative interpretation. Finally, the self-reported nature of the CTADS survey can draw a possibility for information bias.

## Conclusion

Overall, to the best of our knowledge, this study is the first nationally representative data to shed light on the use and characteristics associated with current e-cigarettes exclusively targeted to capture young Canadians’ habits. Illicit drug use approached significance with past 30-day e-cigarette use. Additionally, factors such as using marijuana and alcohol in the past year, having others in the household who smoke, being a current smoker, being male and between the ages of 15–21 were all significantly associated with current e-cigarette smoking in youth. The results of this study further elucidate patterns and risk factors before the TVPA was introduced into legislation, allowing for an accurate baseline which can be used for future studies. Given the increasing popularity of e-cigarettes among young users, this study identifies characteristics which can be carefully observed and easily targeted by public health professionals. With the growing popularity of alcohol, marijuana and illicit drug use among young Canadians, the results from this study, which illustrate the concomitant use of e-cigarettes with these substances, highlight the importance of investigating the possible misuse of e-cigarettes in facilitating substance use. This could be done through surveys such as the CTADS or qualitatively. While it is possible that these trends may decline as the novelty of e-cigarettes and vape pens subsides in the coming years, however, efforts must be made to track use among this cohort closely and implement policy interventions pre-emptively to prevent massive surges in e-cigarette use among youth. While stricter bans on sale of e-cigarettes may seem like the appropriate route, they may not be as efficacious in preventing youth from smoking e-cigarettes [53]. Given that perceptions and beliefs about smoking can play an important role in determining experimentation and habitual e-cigarette smoking behaviors [27], appropriate knowledge dissemination and awareness programs geared towards informing youth about the harms of use, may prove more beneficial. Finally, in light of the role that appealing flavors and designs of e-cigarettes play in determining e-cigarette smoking among youth, restrictions on marketing practices as placed by the TVPA is a step in the right direction. Although it is evident that drug culture is continuously evolving alongside technology, further investigations into reducing hazardous behaviours are needed to prevent the burden of disease from growing among young Canadians.

## Data Availability

The data collected by Statistics Canada as part of the CTADS 2017 can be accessed through a formal application submitted to Statistics Canada via their website at http://www23.statcan.gc.ca/imdb/p2SV.pl?Function=getSurvey&SDDS=4440.

## Abbreviations

CI: Confidence Interval
CTADS: Canadian Tobacco, Alcohol and Drugs Survey
OR: Odds Ratio
TVPA: Tobacco and Vaping Produces Act
